# Supplementary material for: A novel reverse lipase toxin substrate of the Staphylococcus aureus type VII secretion system
Source: Microlife. 2026 Aug 25;7:uqag031. doi: 10.1093/femsml/uqag031 (PMC13560087; doi:10.1093/femsml/uqag031)
Supplement: Supplementary file 1 [file uqag031_supplemental_files.zip › HigginsonSupplementary.pdf]

A novel reverse lipase toxin substrate of the *Staphylococcus aureus* type VII secretion system

Andrew B. Higginson[1], Jasmine Soh[1], Stephen R. Garrett[1], Terry K. Smith[2], Tim R. Blower[3,4] and Tracy Palmer[1]

[1] Newcastle University Biosciences Institute, Newcastle University, Newcastle upon Tyne, NE2 4HH, United Kingdom.

[2] Biomedical Sciences Research Complex, University of St. Andrews, North Haugh, St. Andrews, United Kingdom.

[3] Department of Biosciences, Durham University, Stockton Road, Durham DH1 3LE, United Kingdom;

[4] New England Biolabs, 240 County Road, Ipswich, MA 01938, USA.

e-mail: tracy.palmer@newcastle.ac.uk

SUPPLEMENTARY INFORMATION

Table S1. Bacterial strains used in this study

Strain	Genotype/description	Source/Reference
E. coli		
DH5α	φ80d Δ(*lacZ*)M15 *recA1 endA1 gyrA96 thi-1 hsdR17* (rk-mk+) *supE44 relA1 deoR* Δ(*lacZYA-argF*)U169	Promega
JM110	*rpsL thr leu thi lacY galK galT ara tonA tsx dam dcm glnV44* Δ(*lac-proAB*) e14- [F' *traD36 proAB*+ *lacIq lacZ*ΔM15] *hsdR17*(rK−mK+)	Stratagene
M15 (pREP4)	F-, *lac*, *ara*, *gal*, *mtl*, [Kan^r, *lacI*]	Qiagen
S. aureus		
MRSA252	Wild type	(Holden et al. 2004)
USA300 LAC	Wild type	(Kazakova et al. 2005)
USA300 Δ*essC*	In-frame deletion of *essC* from USA300 LAC	(Garrett et al. 2023)
USA300 Δ*til2*	In-frame deletion of all four *DUF1433* (*til2*) genes in USA300 LAC: three at the *lplII* locus and one at the vSaβ island	This work
USA300 Δ*til2* Δ*essC*	As above, also Δ*essC*	This work
USA300 JE2 NE1540	Tn insertion [Ery^r] in *fakB1* (*SAUSA300_0733*)	NTML (Fey et al. 2013)
USA300 JE2 NE1612	Tn insertion [Ery^r] in prophage tail component (*SAUSA300_1397*)	NTML (Fey et al. 2013)

Table S2. Plasmids used in this study

Plasmid	Description	Source/Reference
pRAB11	*E. coli*/*S. aureus* shuttle vector, inducible expression, Amp^r, Cml^r	(Helle et al. 2011)
pRAB11-TslM$_{pep86}$	pRAB11 encoding TslMpep86 fusion, preceded by *hla* rbs	This work
pRAB11-TslM$_{pep86}$-TlaM	As pRAB11-TslM$_{pep86}$ with TlaM also encoded	This work
pRAB11-TslMLXG$_{pep86}$-TlaM	As pRAB11-TslM$_{pep86}$-TlaM with only the LXG domain of TslM encoded	This work
pRAB11-EsxA$_{pep86}$	pRAB11 encoding EsxA with C-terminal pep86 fusion	(Yang et al. 2024)
pRAB11-TrxA$_{pep86}$	pRAB11 encoding TrxA with C-terminal pep86 fusion	(Yang et al. 2024)
pQE70	Vector for *E. coli*, inducible expression, T5 pro, Amp^r	Qiagen
pQE70-TslMLXG$_{His6}$-TlaM$_{Myc}$	pQE70 encoding the LXG domain of TslM with a C-terminal His6 fusion and TlaM with a C-terminal Myc fusion	This work
pQE70-TslM$_{His6}$-$_{TS}$TlaM-TlaM$_{Myc}$	pQE70 encoding TslM with a C-terminal His6 fusion and two copies of TlaM with an N-terminal TwinStrep fusion and C-terminal Myc fusion	This work
pIMAYZ	*E. coli*/*S. aureus* shuttle vector, temperature sensitive, Cml^r	(Monk & Stinear 2020)
pIMAYZ-til2_lplIII	pIMAYZ carrying *til2* deletion allele for the *lplIII* locus	This work
pIMAYZ-til2_vSaβ	pIMAYZ carrying *til2* deletion allele for the vSaβ island	This work
pIMAY-essC	pIMAY carrying *essC* deletion allele	(Garrett et al. 2023)
pRAB11-TslM-TlaM	pRAB11 encoding TslM-TlaM, preceded by *hla* rbs	This work
pRAB11-TilM-TslM-TlaM	As pRAB11-TslM-TlaM with TilM (full length)	This work
pRAB11-Til2-TslM-TlaM	As pRAB11-TslM-TlaM with NRS_RS09835 (full length)	This work
pRAB11-TslM$_{S171A}$-TlaM	As pRAB11-TslM-TlaM with a S171A substitution in TslM	This work
pRAB11-TslM$_{D226A}$-TlaM	As pRAB11-TslM-TlaM with a D226A substitution in TslM	This work
pRAB11-TslM$_{H261A}$-TlaM	As pRAB11-TslM-TlaM with a H261A substitution in TslM	This work

Table S3. Oligonucleotides used in this study

Construct	Oligonucleotides used for assembly (5'-3')
pRAB11-TslM-TlaM	aaatggatgagagctcgaattcactggc
	attaccctctttatgcataatctggaacatcatatg
	ttatgcataaagagggtaatttatatgagc
	attcgagctctcatccatttatcttcaatgtatttaatag
pIMAYZ-til2_lplIII	aatcgtactagaattcctgcagcccggg
	tctgtaatgagatatcaagcttatcgataccgtcgac
	gcttgatatctcattacagacattaactctc
	caaaaaacataaaaaagaaggccactag
	cttctttttatgtttttgttcattaaagtaactc
	gcaggaattctagtacgatttatagtgcgac
pIMAYZ-til2_vSaβ	ttgagcggtggaattcctgcagcccggg
	aaaataagtagatatcaagcttatcgataccgtcgac
	gcttgatatctacttattttcgttgtcttaattatac
	tttgaataaaaatacgtccaataaataattgttc
	tggacgtatttttattcaaaaaatcaatctccttttc
	gcaggaattccaccgctcaaacttttataaaaag
pQE70-TslMHis6-TSTlaM TlaMMyc	gataaatggatgataattagctgagcttg
	attatctcctagcttatcacaggtcttc
	gtgataagctaggagataatttatatgggatcgggat
	cttttgagcttcccgatcctttttcgaac
	aggatcgggaagctcaaaagatgcaaag
	ctaattatcatccatttatcttcaatgtatttaatag
pRAB11-TslMS171A-TlaM	catgctttaggtgcgtttttag
	tcctaatgggtgaatttcact
pRAB11-TslMD226A-TlaM	gatgctcctattaataatttgaa
	aaaatggccaatattgcg
pRAB11-TslMH261A-TlaM	caagccacgcttaaatcttaca
	accaatcaaaggaaggttagtt
pRAB11-TilM-TslM-TlaM	aattgaataagagctcgaattcactggc
	acctcacttttcatccatttatcttcaatgtatttaatag
	aaatggatgaaaagtgaggtaaaatatgattcg
	attcgagctcttattcaattggacctgaaaag
pRAB11-Til2-TslM-TlaM	gtgctcgatattcaaacccactgag
	agtttatgtgcattgcgaagatcatgg
	ccaccaccaccactaaagagggtaatttatatgagc
	ggtggtggtggtgactaaatagtccaaaaacatcg
pRAB11-TslMpep86	gtttctagttatgaatctttcagacgctc
	cgccggagccactaaatagtccaaaaacatcg
	actatttagtggctccggcgtgagcgg
	aaagattcataactagaaacctcctggtaccatcatactctatcaatg
pRAB11-TslMpep86-TlaM	aaatggatgagaattcactggccgtcgttttac
	attaccctctttagctaatttttttaaacaggcgc

	aattagctaaagagggtaatttatatgagc
	cagtgaattctcatccatttatcttcaatgtatttaatag
pRAB11-TsIMLXGpep86-TlaM	tctagttatgatctctgttaaaaaaaccaaaag
	taacagagatcataactagaaacctcctg

Supplementary References

Fey PD, Endres JL, Yajjala VK et al. A genetic resource for rapid and comprehensive phenotype screening of nonessential *Staphylococcus aureus* genes. *MBio*, 2013;4(1):e00537-12.

Garrett S R, Mietrach N, Deme J et al. A type VII-secreted lipase toxin with reverse domain arrangement. *Nat Commun* 2023;14(1):1–16.

Helle L, Kull M, Mayer S et al. Vectors for improved Tet repressor-dependent gradual gene induction or silencing in *Staphylococcus aureus*. *Microbiology* 2011;157(12):3314–23.

Holden MT, Feil EJ, Lindsay JA et al. Complete genomes of two clinical *Staphylococcus aureus* strains: Evidence for the evolution of virulence and drug resistance. *Proc Natl Acad Sci USA* 2004;101(26):9786–91.

Kazakova SV, Hageman JC Matava M et al. A clone of methicillin-resistant *Staphylococcus aureus* among professional football players. *New Eng J Med* 2005;352(5):468–75.

Kläui AJ, Boss R and Graber HU. Characterization and comparative analysis of the *Staphylococcus aureus* genomic Island vSaβ: an in silico approach. *J Bacteriol* 2019;201(22):e00777-18.

Monk IR and Stinear TP. From cloning to mutant in 5 days: rapid allelic exchange in *Staphylococcus aureus*. *Access Microbiol* 2020;3(2):000193.

Yang Y, Scott AA, Kneuper H et al. High-throughput functional analysis provides novel insight into type VII secretion in *Staphylococcus aureus*. *Open Biol* 2024;14(8):240060.

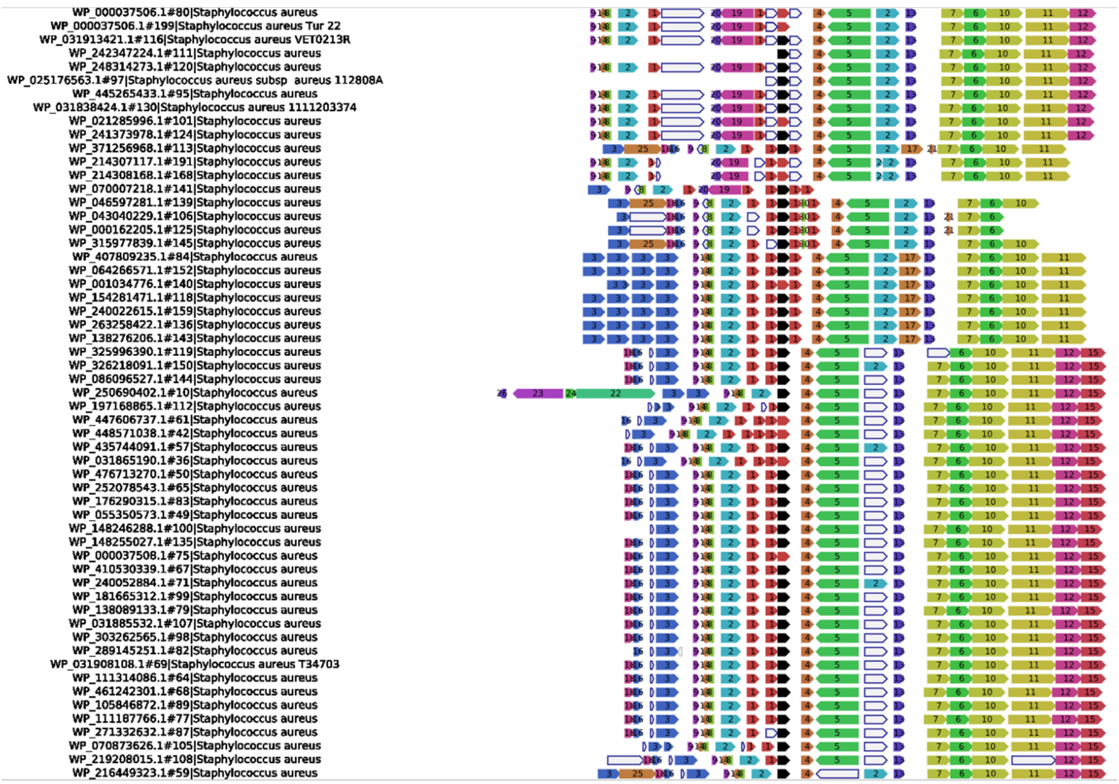

Figure S1. Gene neighbourhood analysis of DUF1433-encoding genes. The DUF1433 protein WP_001034756.1 was used in a webFlags search of *S. aureus* genomes present in RefSeq to identify where homologous proteins were encoded. From the output, genes shaded black or red (numbered 1) correspond to DUF1433 (Til2)-encoding genes. Genes shaded blue and numbered 3 correspond to *til1* (encoding immunity proteins to TslB), genes shaded orange and numbered 25 correspond to *tslB,* genes shaded pink and numbered 19 encode the Tsl2 family of reverse phospholipases (TslO in this instance) and genes shaded purple and numbered 20 encode Tla2 proteins (TlaO in this instance). White genes with a blue outline are pseudogenes.

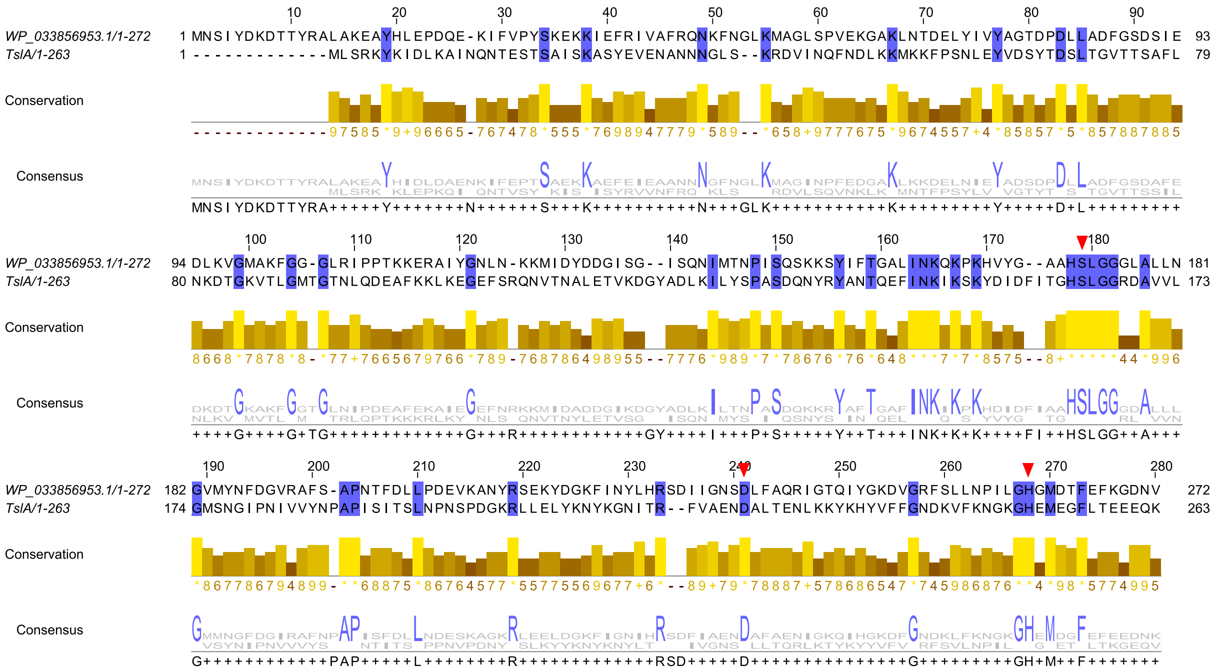

Figure S2. Alignment of the phospholipase domains of TslO (WP_033856953.1) and TslA. Pairwise alignment performed with Smith-Waterman algorithm, visualised in Jalview. Blue residues/logo indicate residue matches. Phospholipase catalytic triad of TslA (S164, D224, H251) and aligning predicted catalytic triad of TslO shown with red arrows.

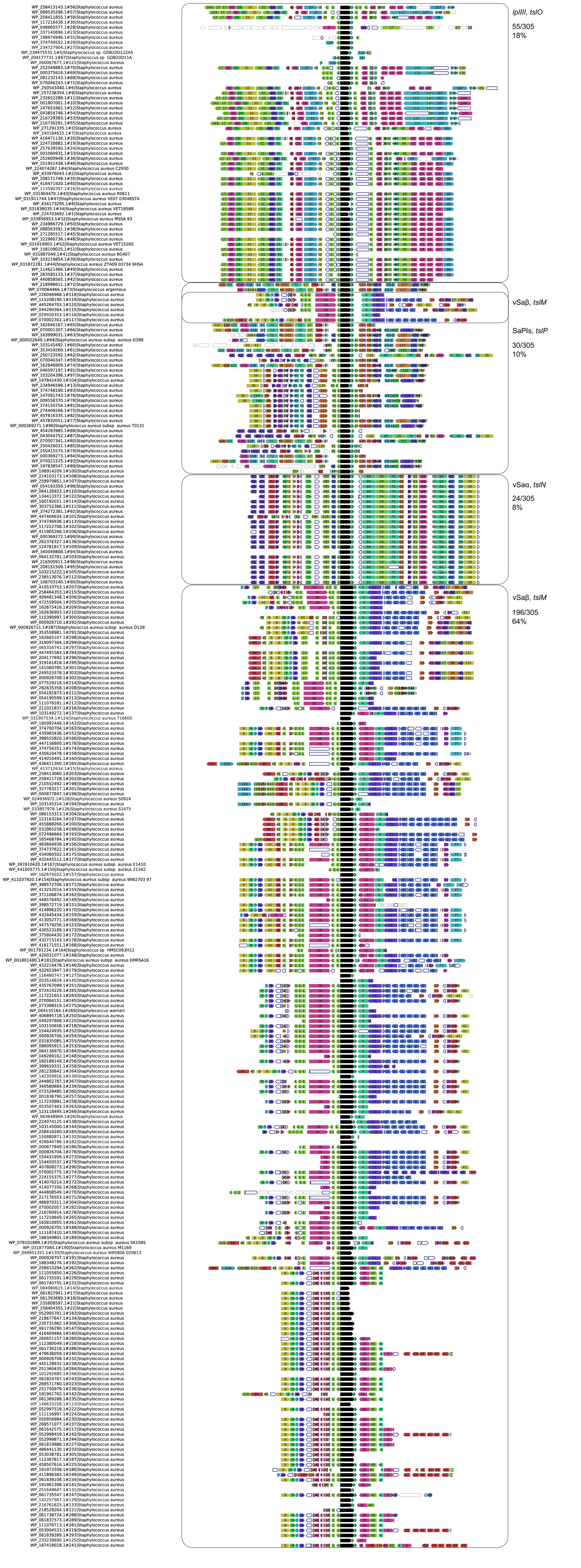

lplIII, *tslO*
55/305
18%
vSaβ, *tslM*
SaPIs, *tslP*
30/305
10%
vSaα, *tslN*
24/305
8%
vSaβ, *tslM*
196/305
64%

Figure S3. Gene neighbourhood analysis of novel phospholipase-encoding genes. The protein sequence of WP_033856953.1, a putative phospholipase, was used in a FlaGs search of *S. aureus* genomes present in RefSeq to identify where homologous proteins were encoded. From the output, genes shaded black correspond to Til2 lipase-encoding genes. The TslM, TslN, TslO and TslP-encoding families are indicated, alongside their chromosomal loci.

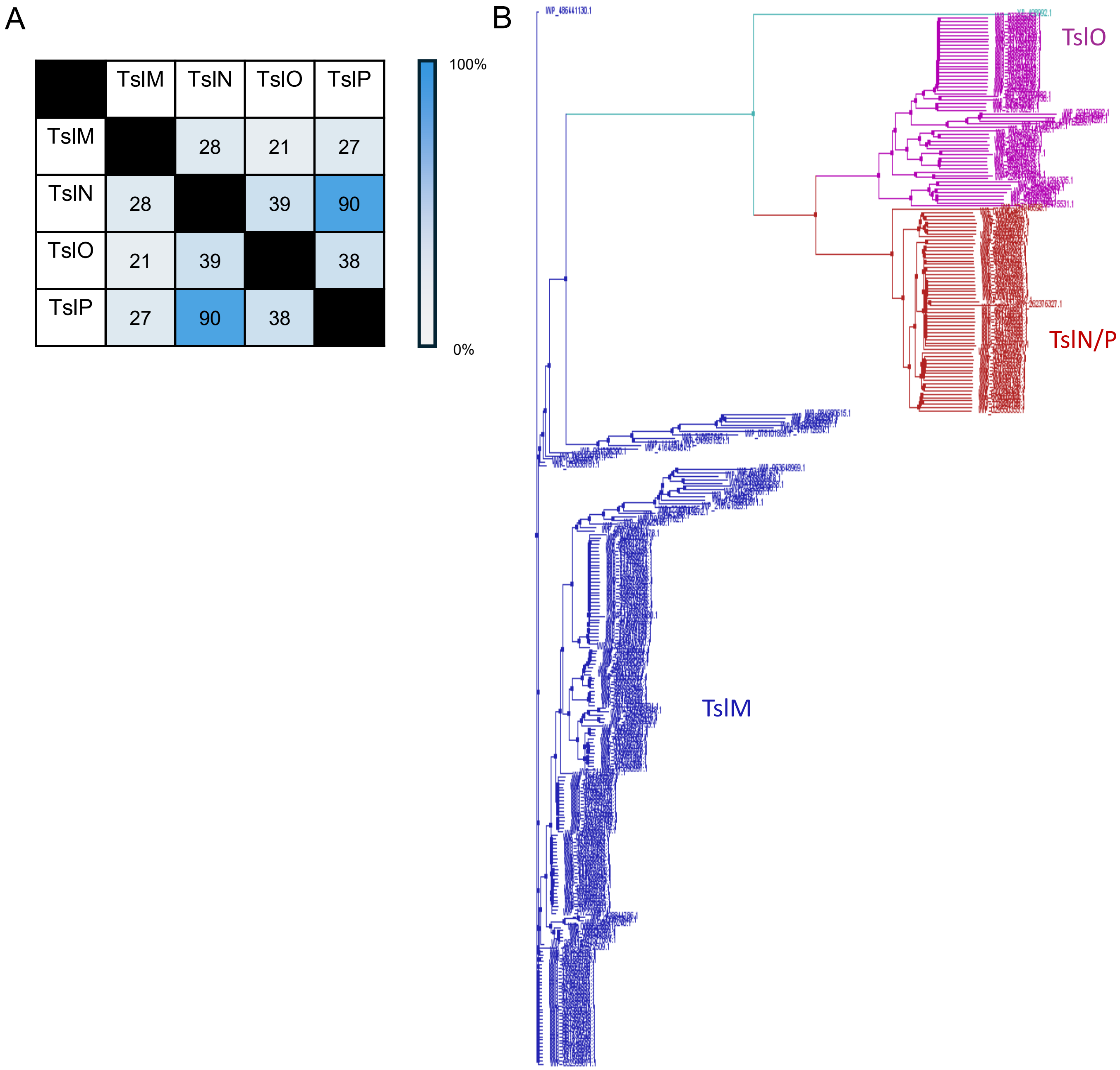

Figure S4. A. Protein sequence identity matrix of the four representative members of the Tsl2 family. Seq. ID was calculated by pairwise alignment. B. Maximum likelihood phylogenetic tree of the 305 Tsl2 protein sequences identified by BLAST search, as well as TslA. Families are coloured as follows: TslM (blue), TslO (purple), TslN/P (red), TslA (cyan). TslA groups with the smaller homologues, likely due to its relative size.TslP and TslN have high sequence similarity and are distinguishable by their locus, with *tslP* being encoded on SaPIs.

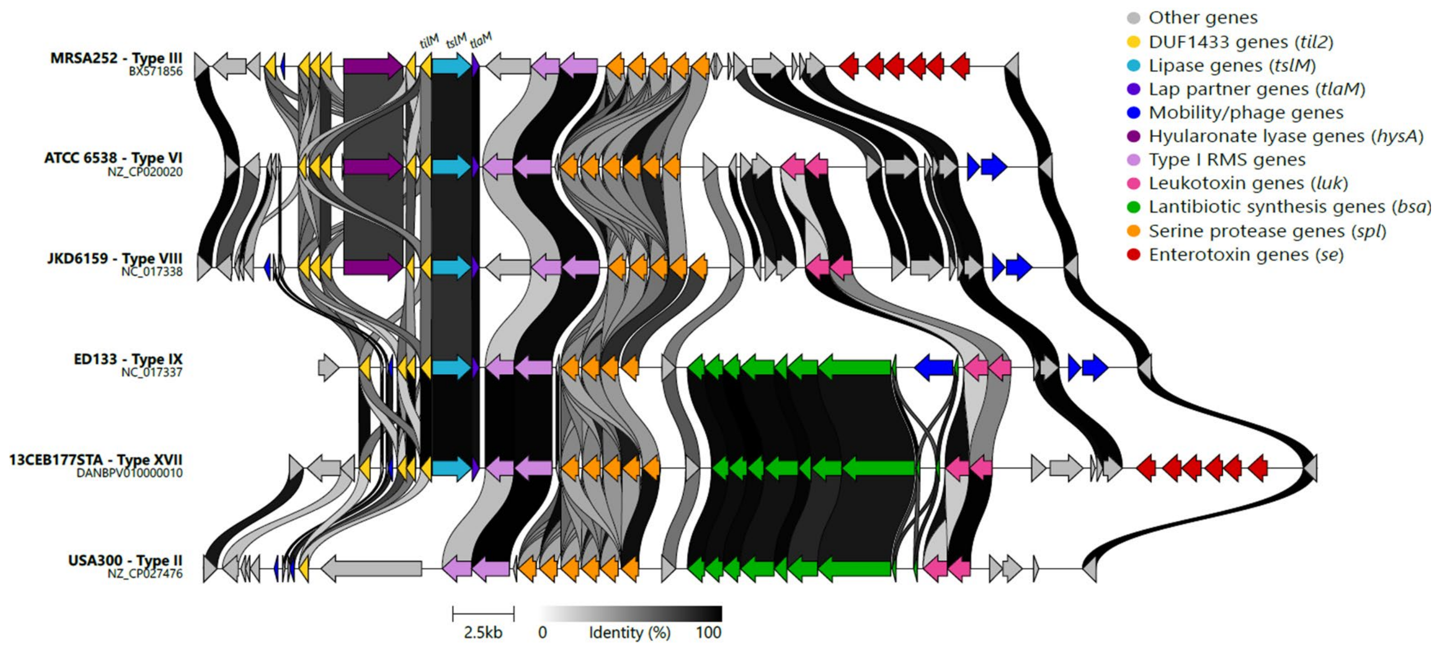
MRSA252 - Type III
BX571856
ATCC 6538 - Type VI
NZ_CP020020
JKD6159 - Type VIII
NC_017338
ED133 - Type IX
NC_017337
13CEB177STA - Type XVII
DANBPV010000010
USA300 - Type II
NZ_CP027476
tilM
tslM
tlaM
Other genes
DUF1433 genes (til2)
Lipase genes (tslM)
Lap partner genes (tlaM)
Mobility/phage genes
Hylaronate lyase genes (hysA)
Type I RMS genes
Leukotoxin genes (luk)
Lantibiotic synthesis genes (bsa)
Serine protease genes (spl)
Enterotoxin genes (se)
2.5kb
0
Identity (%)
100

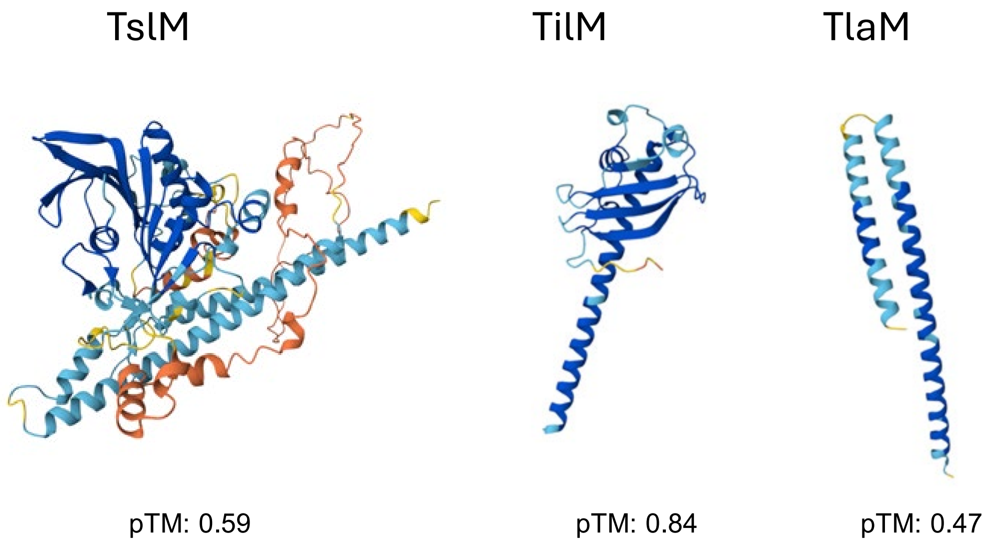

Figure S6. Alphafold3 models of TslM, TilM and TlaM coloured by confidence (where red is low confidence and blue high confidence). Predicted Template Modeling scores (pTM) are also given.

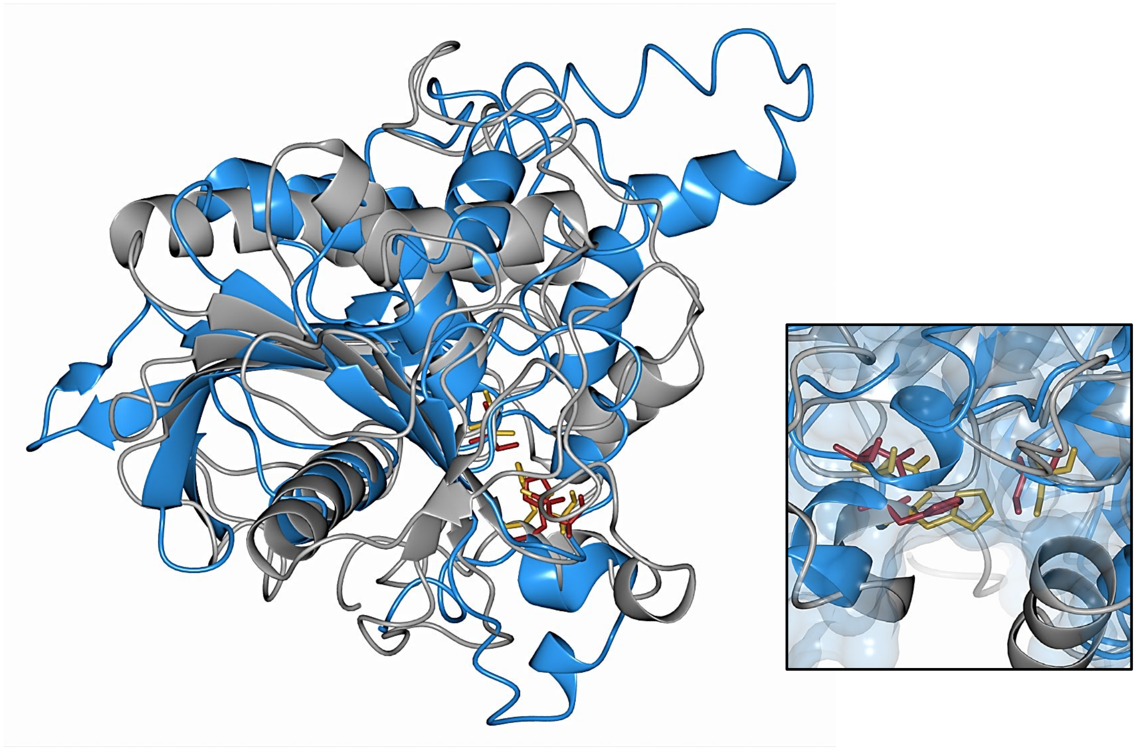

Figure S7. Lip2 and TslM superposition. Superposition of the lipase domain of TslM (residues 1-268, blue) and Lip2, a characterised lipase (PDB ID: 3o0d(Bordes et al., 2010), grey). The predicted catalytic triad of TslM (S171A, D226A, H261A) is shown in red, and the catalytic triad of Lip2 (S162, D230, H289) in gold. Inset: close up view of the predicted active site of TslM superposed with that of Lip2, with surface view shown. Superposition was performed with the SSM method.

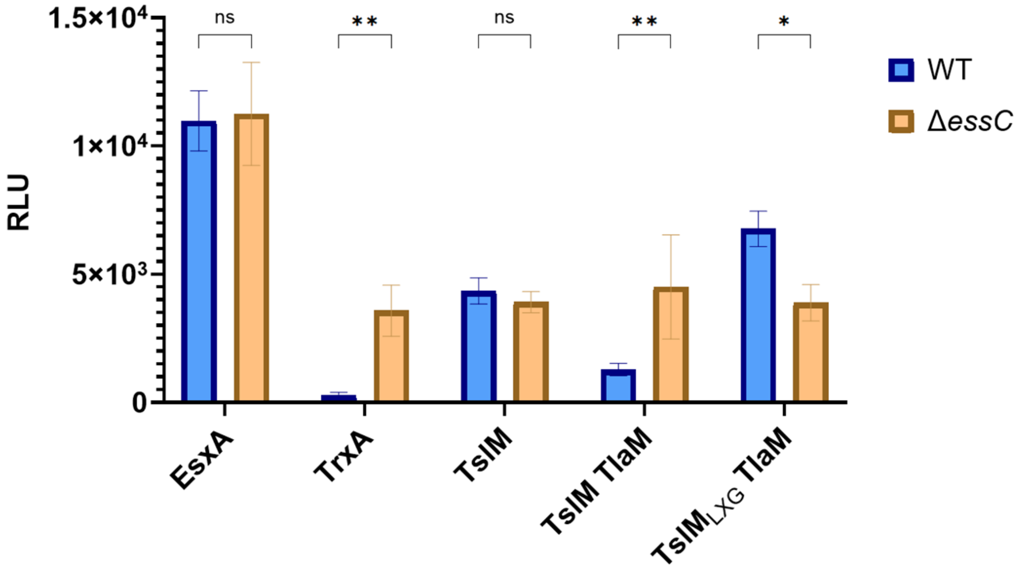

Figure S8. Nanoluciferase assay of cytoplasmic fraction of samples from Figure 4. Relative luminescence of cytoplasmic fraction from cultures producing C-terminal pep86-tagged TslM alone, or with TlaM, or a pep86-tagged C-terminal domain alongside TlaM ($TslM_{LXG}$-TlaM) in both wild type (blue) and Δ*essC* (orange) strains. C-terminally pep86-tagged EsxA (T7SS-secreted substrate) and TrxA (cytoplasmic protein) were included as controls. Readings were taken at maximum luminescence which reached 7 minutes following the addition of luciferase substrate and 11S. Error bars show standard deviation of three biological replicates. Significant difference was determined by 2way ANOVA: P values represented by asterisks: * <0.05, ** <0.01.

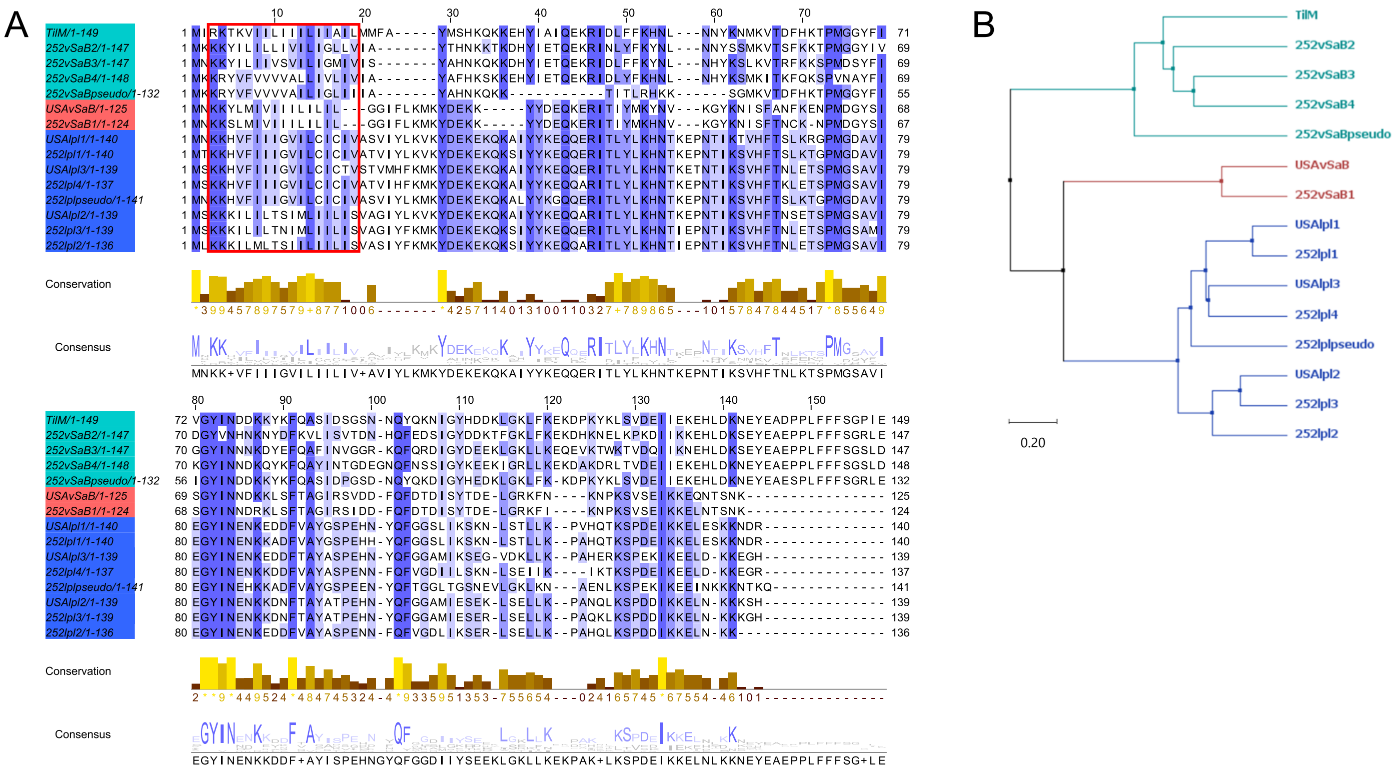

Figure S9. Multiple sequence alignment of the Til2 proteins encoded by USA300 and MRSA252. A. Alignment performed with the Muscle algorithm, visualised in Jalview. Residues and logo coloured by sequence conservation. til2 genes are numbered from the first gene in a downstream direction in their respective loci: vSaβ or lpllll. Pseudogenes (indicated) were repaired for the purposes of the alignment by the manual removal of frameshifts and subsequent translation to the shown amino acid sequence. The conserved N-terminal transmembrane helix is boxed in red. Sequences are coloured and grouped according to the tree shown in B. B. Maximum likelihood phylogeny generated from the 15 Til2 proteins encoded in USA300 and MRSA252. Three clear clades emerge, with TilM grouping best with the other Til2 proteins encoded at the MRSA252 vSaβ locus, and all lplll encoded Til2 proteins from both strains grouping together. This indicates that immunity protein diversity is locus specific, possibly reflecting the specific evolution of immunities required to protect against toxin subtypes within each toxin family. NRS_RS09835, used as the noncognate Til2 in toxicity experiments of Fig 4D, is labelled here as USAvSaβ.

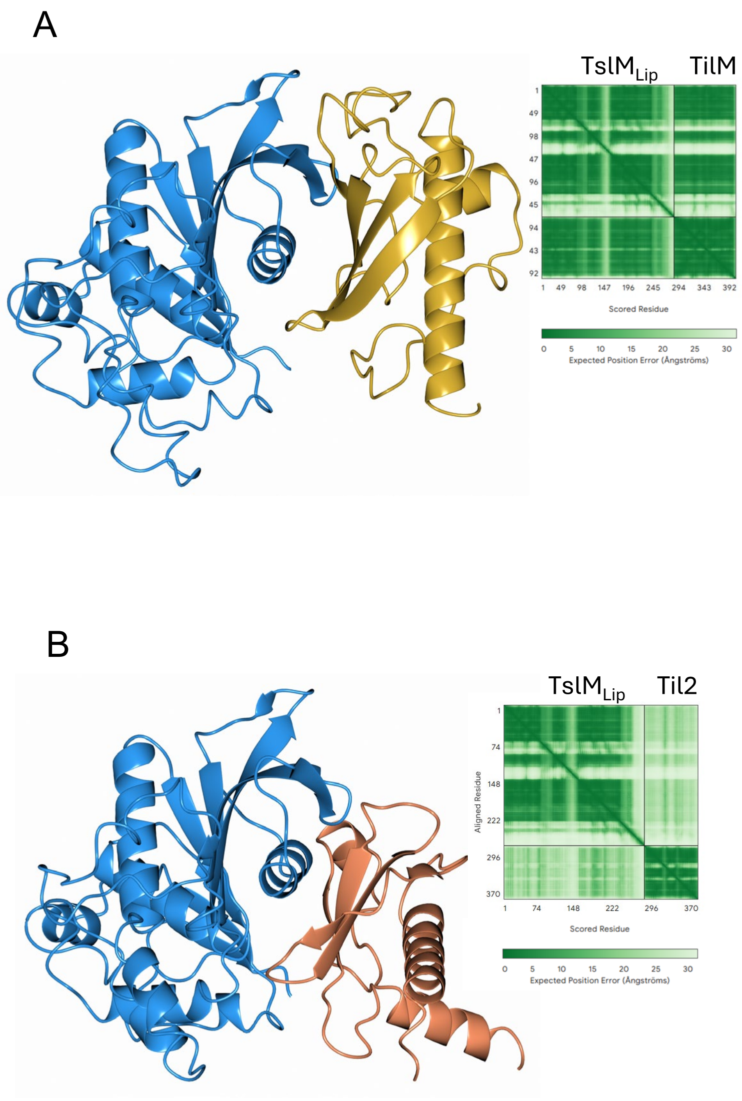

Figure S10. Modelling of Til2 binding to TslM. A. TslM lipase domain (blue) and TilM (without TM helix, gold) complex generated by AlphaFold3. Inset: Predicted Aligned Error plot of the complex generated by AlphaFold3, iPTM = 0.89. B. TslM lipase domain (blue) and noncognate Til2 NRS_RS09835 (without TM helix, orange) complex generated by AlphaFold3. Inset: Predicted Aligned Error plot of the complex generated by AlphaFold3, iPTM = 0.23. Note the difference in predicted binding position and ipTM confidence scores between the complexes.
